# Effect of *Bifidobacterium bifidum* G9-1 on the Intestinal Environment and Diarrhea-Predominant Irritable Bowel Syndrome (IBS-D)-like Symptoms in Patients with Quiescent Crohn’s Disease: A Prospective Pilot Study

**DOI:** 10.3390/jcm12103368

**Published:** 2023-05-09

**Authors:** Toshihiko Tomita, Hirokazu Fukui, Takuya Okugawa, Takashi Nakanishi, Masatoshi Mieno, Keisuke Nakai, Hirotsugu Eda, Yoshitaka Kitayama, Tadayuki Oshima, Shinichiro Shinzaki, Hiroto Miwa

**Affiliations:** Division of Gastroenterology and Hepatology, Department of Internal Medicine, Hyogo Medical University, Nishinomiya 663-8501, Japan; tomita@hyo-med.ac.jp (T.T.); okugawat@hyo-med.ac.jp (T.O.); ta-nakanishi@hyo-med.ac.jp (T.N.); ma-mieno@hyo-med.ac.jp (M.M.); k-nakai@hyo-med.ac.jp (K.N.); eda@hyo-med.ac.jp (H.E.); yoshitaka591027@hyo-med.ac.jp (Y.K.); t-oshima@hyo-med.ac.jp (T.O.); sh-shinzaki@hyo-med.ac.jp (S.S.);

**Keywords:** Crohn’s disease, irritable bowel syndrome, probiotics, inflammation, anxiety, gut microbiome

## Abstract

Diarrhea-predominant irritable bowel syndrome (IBS-D)-like symptoms are distressing for patients with quiescent Crohn’s disease (qCD) and worsen their quality of life. In the present study, we assessed the effect of the probiotic *Bifidobacterium bifidum* G9-1 (BBG9-1) on the intestinal environment and clinical features in patients with qCD. Eleven patients with qCD, who met the Rome III diagnostic criteria for IBS-D, received BBG9-1 (24 mg) orally three times daily for 4 weeks. Indices of the intestinal environment (fecal calprotectin level and gut microbiome) and clinical features (CD/IBS-related symptoms, quality of life and stool irregularities) were evaluated before and after treatment. Treatment with BBG9-1 tended to reduce the IBS severity index in the studied patients (*p* = 0.07). Among gastrointestinal symptoms, abdominal pain and dyspepsia tended to be improved by the BBG9-1 treatment (*p* = 0.07 and *p* = 0.07, respectively), and IBD-related QOL showed a significant improvement (*p* = 0.007). With regard to mental status, the patient anxiety score was significantly lower at the endpoint of BBG9-1 treatment than at the baseline (*p* = 0.03). Although BBG9-1 treatment did not affect the fecal calprotectin level, it suppressed the serum MCP-1 level significantly and increased the abundance of intestinal *Bacteroides* in the study patients. The probiotic BBG9-1 is able to improve IBD-related QOL with a reduction of anxiety score in patients with quiescent CD and IBS-D-like symptoms.

## 1. Introduction

Although the etiology of Crohn’s disease (CD) is still unclear, treatment for CD patients has greatly improved due to the development of biologic agents, resulting in a high rate of quiescent remission [1]. Although, as a result, the number of patients with quiescent CD (qCD) has increased worldwide, their quality of life (QOL) is never equivalent to that in healthy individuals [2]. Indeed, patients with qCD frequently have symptoms that are characteristic of diarrhea-predominant irritable bowel syndrome (IBS-D) [2,3], associated with deterioration of their QOL [4]. Since a diagnosis of IBS requires the absence of any organic diseases [5], patients with qCD are never diagnosed as having IBS even if their abdominal symptoms meet the diagnostic criteria. Accordingly, the symptoms that meet the diagnostic criteria for IBS-D in patients with qCD have been named as IBS-D-like symptoms [2,3,6].

It is noteworthy that a large group (30–50%) of patients with qCD have IBS-like symptoms [2,3,4,7] although the mechanism underlying such symptoms has remained unclear. In this context, we and others have recently suggested that various factors including intestinal microinflammation, the gut microbiome [6,8,9,10] and psychological stress [4,10,11,12] commonly play a pivotal role in the pathophysiology of both IBS and qCD. However, therapeutic strategies for IBS-D-like symptoms in quiescent CD remain to be established. In the present study, we investigated the effect of the probiotic *Bifidobacterium bifidum* G9-1 (BBG9-1) on the intestinal environment and clinical features in patents with qCD.

## 2. Materials and Methods

### 2.1. Patients

Patients who had qCD with IBS-D-like symptoms were enrolled into this prospective intervention study at our university hospital between March and October 2021. The protocol was designed according to the Declaration of Helsinki, approved by the Japan Physicians Association Institutional Review Board (Approval No. CRB3190007) and registered in the Japan Registry of Clinical Trials (jRCT) (Registration No. jRCTs 031200327). Similarly to previous work [3,6,10,13,14], qCD was defined as a CD activity index (CDAI) of ≤150 with a C-reactive protein (CRP) level of ≤0.3 mg/dL. Before this study, mucosal healing had been confirmed by colonoscopy and/or capsule endoscopy. IBS-D-like symptoms were assessed by a Japanese version of the Rome III diagnostic questionnaire for functional gastrointestinal disorders [5,15].

Patients who met all of the conclusion criteria for both qCD and IBS-D were enrolled. Clinical data and symptoms were assessed by a self-completed symptom questionnaire. Patients satisfying the inclusion and exclusion criteria (Supplementary Table S1) were registered, and all of them provided written informed consent before participating.

### 2.2. Study Design

**Figure 1:**
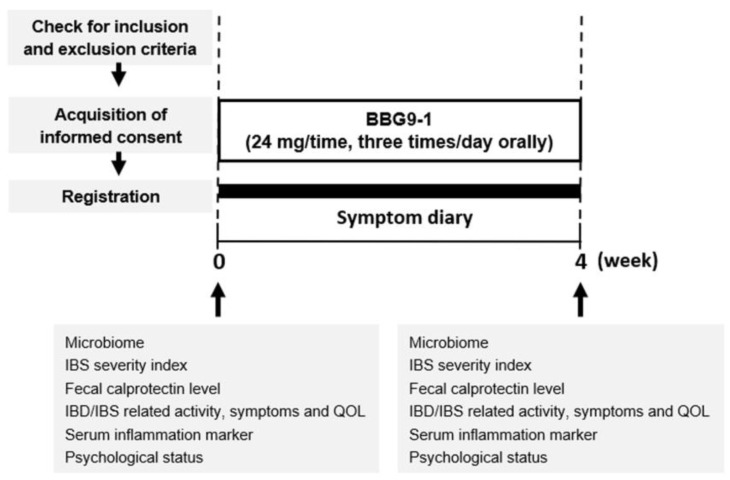
Study design. BBG9-1, *Bifidobacterium bifidum* G9-1; IBD, inflammatory bowel disease; IBS, irritable bowel syndrome; QOL, quality of life.

This prospective open label intervention study comprised a 4-week period of treatment with the probiotic BBG9-1 (Figure 1). Twelve patients who met criteria for quiescent CD with IBS-D-like symptoms were registered, but one of them later withdrew consent and dropped out before treatment. The remaining eleven enrolled patients were subsequently treated with BBG9-1 (24 mg/time, three times/day orally) for 4 weeks. The characteristics of the patients including age, sex, BMI, disease duration, current medications, prior surgery, CRP level and CD activity index are presented in Table 1. Six of 11 patients had been treated with 5-ASA although 5-ASA is currently becoming no more a treatment for CD [16]. Although 10 of 11 patients had undergone elemental diet, no significant relationship has been confirmed between elemental diet and IBS-D-like symptoms in qCD patients in our previous research [10]. When starting and ending the treatment, blood and stool samples were obtained from all the participants, as well as their answers to a questionnaire. During the treatment period, the patients recorded their medication status and symptoms including stool consistency and frequency on a diary paper file every day. A master list connecting to ID numbers for the study patients was securely managed by the researchers in charge.

The primary endpoint was the change in the fecal calprotectin level at the end of the treatment relative to that at the start. The secondary endpoint was the changes in serum inflammation markers and IBD/IBS-related activity, symptoms and QOL at the end of the treatment relative to the values at the start.

### 2.3. Questionnaires

See “Materials and Methods” in reference [10] and associating references [17,18,19,20,21,22,23,24].

### 2.4. Measurement of the Fecal Calprotectin Level

See “Materials and Methods” in reference [10].

### 2.5. Analysis of the Gut Microbiome

See “Materials and Methods” in reference [25].

Microbiome diversity was assessed using the Shannon index, Faith PD (phylogenetic diversity), and observed OTUs (operational taxonomic units) based on 97% nucleotide sequence identity. Comparison of each taxon in the gut microbiota was performed at the genus level.

### 2.6. Statistical Analyses

All statistical analyses were carried out using SAS version 9.4 (SAS Institute, Cary, NC, USA). Results are shown as means ± SD or median and inter-quartile range. Paired *t* test was applied to compare clinical data, questionnaire scores, serum cytokines level, fecal calprotectin level and microbiota diversity. Wilcoxon signed-rank test was applied for comparison of microbiome abundance. Statistical significance was *p* < 0.05.

## 3. Results

### 3.1. Effect of BBG9-1 on CD Activity Index and IBS Severity Index in Patients with Quiescent CD Patients and IBS-D-like Symptoms

**Figure 2:**
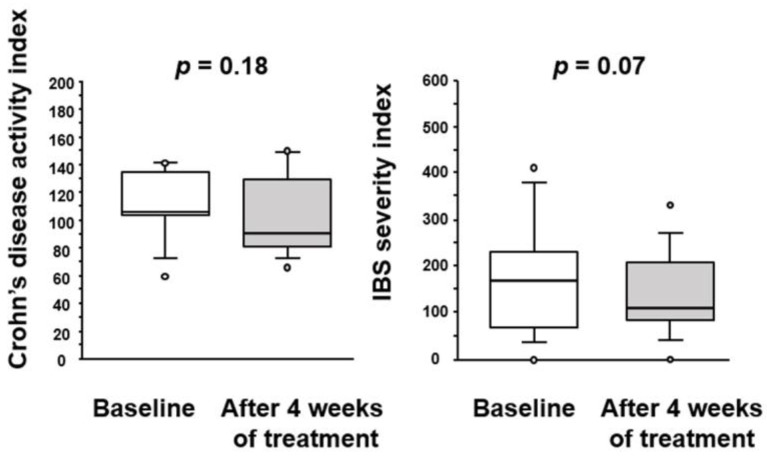
Effect of BBG9-1 on the CD activity index and IBS severity index in patients with qCD and IBS-D-like symptoms. Results are shown as median and inter-quartile range. White dot is an outlier.

After treatment with BBG9-1, the CD activity index was decreased (median 90.1 [79.0–130.2]) relative to that at the baseline (106.1; [103.7–139.4]), but not to a significant degree. The IBS severity index was decreased almost significantly (*p* = 0.07) from 170 [60–240] to 110 [80–220] (Figure 2).

### 3.2. Effect of BBG9-1 on Gastrointestinal Symptoms in Patients with Quiescent CD and IBS-D-like Symptoms

**Figure 3:**
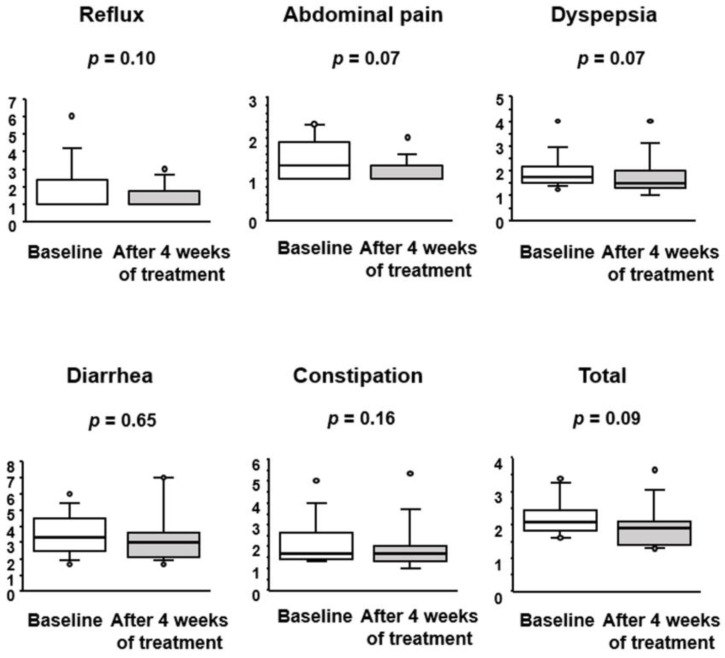
Effect of BBG9-1 on gastrointestinal symptoms in patients with qCD and IBS-D-like symptoms. Results are shown as median and inter-quartile range. White dot is an outlier.

Gastrointestinal symptoms including reflux, abdominal pain, dyspepsia, diarrhea and constipation was evaluated using a gastrointestinal symptom rating scale (Figure 3). Overall, gastrointestinal symptoms showed a marked tendency for improvement by BBG9-1 treatment (*p* = 0.09). The observed improvements in abdominal pain and dyspepsia reached an almost significant level (*p* = 0.07 and *p* = 0.07, respectively).

### 3.3. Effect of BBG9-1 on Disease-Specific and Health-Related Quality of Life in Patients with Quiescent CD and IBS-D-like Symptoms

**Figure 4:**
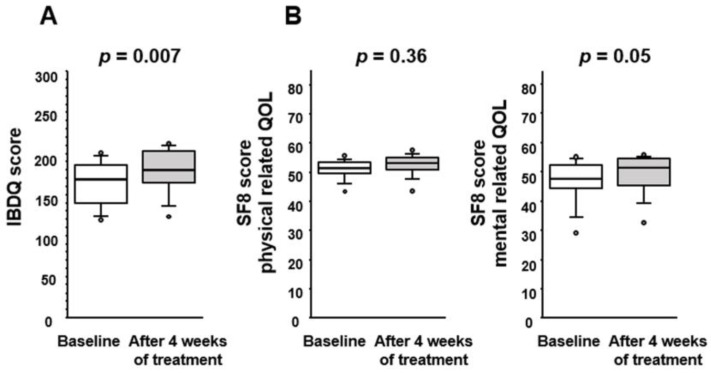
Effect of BBG9-1 on (**A**) the IBD questionnaire (IBDQ) score and (**B**) 8-item short-form health survey (SF-8) scores for patients with qCD and IBS-D-like symptoms. Results are shown as median and inter-quartile range. White dot is an outlier.

We next assessed the effect of the probiotic BBG9-1 on the QOL of patients with qCD and IBS-D-like symptoms (Figure 4). The IBDQ score after the treatment (180 [164–204]) was significantly better than that at the baseline (169 [133–189]), suggesting that disease-specific QOL had been improved by BBG9-1 (Figure 4A). We also evaluated the general QOL of these patients using the SF-8 health survey (Figure 4B). Although the physical summary score after BBG9-1 treatment did not differ from that before treatment, the post-treatment mental summary score (51.4 [44.8–54.6]) was almost significantly higher than that at the baseline (47.7 [44.1–52.7], *p* = 0.05).

### 3.4. Effect of BBG9-1 on Depression and Anxiety Status in Patients with Quiescent CD and IBS-D-like Symptoms

**Figure 5:**
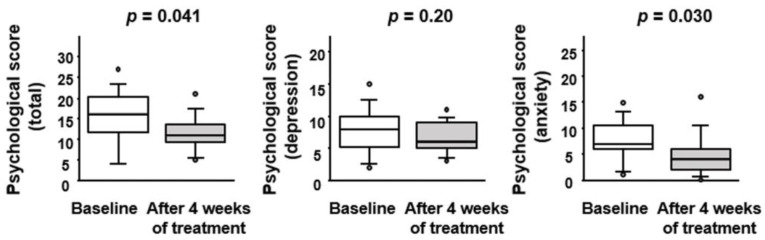
Effect of BBG9-1 on psychological scores in patients with qCD and IBS-D-like symptoms. Results are shown as median and inter-quartile range. White dot is an outlier.

Using the HADS questionnaire, we evaluated in more detail the mental status of patients with qCD and IBS-D-like symptoms (Figure 5). Although the depression score did not differ between the baseline (8 [5–10]) and the endpoint of BBG9-1 treatment (6 [5–9]), the anxiety score was significantly lower at the endpoint (4 [2–6]) than at the baseline (7 [6–11]). The total HADS score was significantly lower at the endpoint (11 [9–14]) than at the baseline (16 [11–21]).

### 3.5. Effect of BBG9-1 on Fecal Calprotectin and Serum Cytokines Levels in Patients with Quiescent CD and IBS-D-like Symptoms

**Figure 6:**
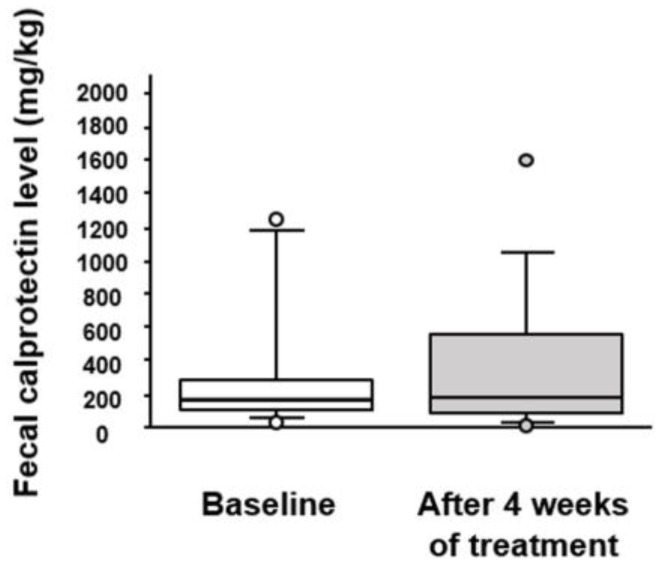
Effect of BBG9-1 on the level of fecal calprotectin in patients with qCD and IBS-D-like symptoms. Results are shown as median and inter-quartile range. White dot is an outlier.

To assess the change in the inflammatory condition of the intestinal environment in patients with qCD and IBS-D-like symptoms, we measured the level of fecal calprotectin (Figure 6). This revealed that the fecal calprotectin level before BBG9-1 treatment (165.0 [94.6–305.0] mg/kg) did not differ significantly from that after treatment (185.0 [86.0–644.0] mg/kg).

We also examined the levels of serum cytokines as inflammatory markers in the study patients. As shown in Table 2, the level of serum MCP-1 (monocyte chemotactic protein chemotactic protein 1) was significantly decreased by the BBG9-1 treatment. In addition, the levels of IL-8 and MIP-1b (macrophage inflammatory protein 1b) tended to be decreased as a result of the treatment.

### 3.6. Effect of BBG9-1 on the Fecal Microbiome in Patients with qCD and IBS-D-like Symptoms

**Figure 7:**
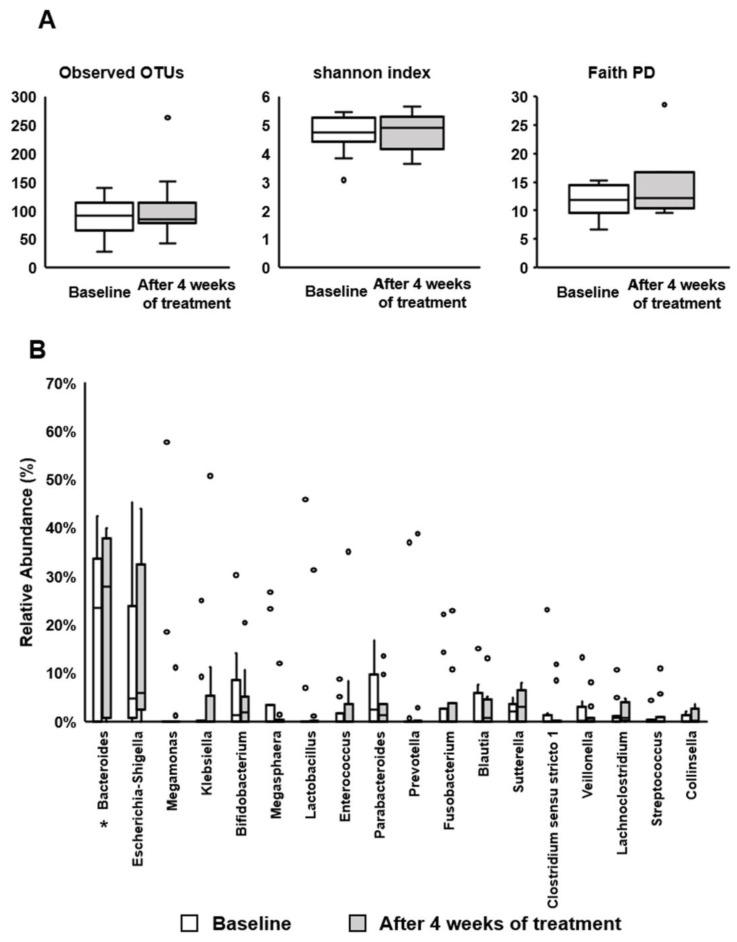
Effect of BBG9-1 on the gut microbiome profile in patients with qCD and IBS-D-like symptoms. (**A**) Diversity of the gut microbiome. (**B**) Relative abundance of intestinal bacteria at the genus level. Results are shown as median and inter-quartile range. White dot is an outlier. Significant differences between the baseline and after 4 weeks of treatment at * *p* < 0.05. OTUs, operational taxonomic units; PD, phylogenetic diversity.

Among indices of gut microbiome diversity, the PD tended to be increased after treatment with BBG9-1 (*p* = 0.08) (Figure 7A). The Shannon index and observed OTUs after treatment did not differ relative to the values before treatment (Figure 7A).

Furthermore, we investigated the gut microbiome profile at the genus level before and after BBG9-1 treatment in the study patients (Figure 7B). We found that the treatment significantly increased the abundance of Bacteroides in the patients (*p* = 0.049).

## 4. Discussion

As the number of patients with qCD has been increasing due to advances in medication, the management for those patients is gathering attentions. As IBS-D-like symptoms certainly disturb the QOL of such patients [2,26], it is important to clarify the pathophysiology of such symptoms and establish a form of effective management. The profile of the gut microbiome has recently attracted much attention because its imbalance (dysbiosis) plays an important role in the pathophysiology of not only IBD/IBS but also various systemic diseases [27,28]. In this context, probiotics that can correct dysbiosis and the intestinal environment would be potentially useful, and in fact several studies have reported that probiotics show promise for the treatment of IBD or IBS [29,30,31]. However, most of those studies tried a combination of probiotic strains, and very few tested a single strain [31,32,33]. It is also noteworthy that no effective therapeutic management for IBS-D-like symptoms in qCD has yet been established, although the pathophysiological mechanism of such symptoms likely differs from that in diagnostically confirmed IBS patients without organic disease. In this context, it is noteworthy that we administered a single probiotic strain (BBG9-1; *Bifidobacterium bifidum*) to patients with qCD and IBS-D-like symptoms and found that it improved their IBD-related QOL and relieved their abdominal pain. Moreover, although not to a statistically significant degree, the severity of IBS was markedly reduced by the treatment. These findings suggest that the probiotic BBG9-1 would be a promising for treatment of patients with qCD and IBS-D-like symptoms.

It still remains unclear how BBG9-1 ameliorates IBS-D-like symptoms and improves QOL. In general, it is well known that clinical symptoms and QOL is likely affected by placebo effect [34]. On the other hand, we have recently suggested that minimal inflammation in the intestine is correlated with the development of IBS-D-like symptoms in patients with qCD [10]. Therefore, we decided the primary endpoint as the change in the fecal calprotectin level, which is an objective marker, at the end of the treatment relative to that at the start. As a result, we found that BBG9-1 did not change the level of fecal calprotectin in patients with qCD and IBS-D-like symptoms statistically. This finding may indicate that minimal inflammation is not associated with the IBS-D-like symptoms in qCD, but we never exclude the possible link between minimal inflammation and IBS-D-like symptoms. In the present study we evaluated fecal calprotectin level at the start and endpoint of treatment alone. In addition, the calprotectin test is very sensitive and might be greatly changed during the treatment period. Thus, in order to investigate the relationship between fecal calprotectin level and clinical data more precisely, we should evaluate them at various timepoint during this small-scale study. On the other hand, it was interesting that the serum levels of proinflammatory cytokines, including MCP-1, IL-8 and MIP-1b, were decreased after the treatment with BBG9-1, possibly indicating inhibition of systemic inflammation. Indeed, the anti-inflammatory effect of BBG9-1 has been supported in studies using an animal model of drug/stress-induced intestinal inflammation [35,36,37]. Therefore, we believe that this effect of BBG9-1 should be assessed in a large-scale human clinical trial.

In this study, we showed that BBG9-1 treatment greatly improved the QOL of patients with qCD and IBS-D-like symptoms in term of psychological anxiety. It is well known that mental stress plays a key role in the pathophysiology of IBS [38]. Recent studies have revealed that patients with quiescent CD and IBS-D-like symptoms have significantly greater anxiety than those without [4,10,12]. From this viewpoint, it is interesting that BBG9-1 treatment greatly improved psychological anxiety in the present patients. There has been accumulating evidences to suggest that interaction between the intestinal environment and psychological stress can explain the pathophysiology of IBS [39]. In the present study patients, we found that the abundance of *Bacteroides* was significantly increased after the treatment with BBG9-1. Interestingly, *Bacteroides* has been reported to influence depression and anxiety by modulating the function of GABA [40]. Moreover, it is likely that BBG9-1 plays a role in suppressing the hypothalamic-pituitary-adrenal reaction to psychological stress [35]. Although it remains to be clarified how these strains act on the brain-gut axis, we speculate that they may exert beneficial effects for suppression of psychological stress.

We have additionally reported interesting findings that serum level of total cholesterol and HDL was significantly decreased in qCD patients with IBS-D-like symptoms after BBG9-1 treatment (Supplementary Table S2). There is an interaction between the microbiome and bile composition and the bile synthesis [41,42]. Furthermore, several papers reported that BA changing treatments is benefitable in IBD patients [43,44]. In these contexts, it is very interesting that BBG9-1 improved IBD-related QOL with a reduction of serum cholesterol. Of note, it is likely that not only *Bifidobacterium* but also *Bacteroides* strains play a role in the reduction of serum cholesterol level [45,46]. The mechanism by which those strains reduce the serum cholesterol level is unclear. However, it has been speculated that those strains are possible to inhibit the synthesis of cholesterol using their producing short-chain fatty acids (especially butyrate and acetic acid) [45,47]. Importantly, gut microbiome plays a pivotal role in the bile acid metabolism including deconjugation of primary bile acid using bile salt hydrase and absorption of lipid in the ileum [41,42]. Furthermore, it has been well known that imbalance of bile acid metabolism is occurred in IBD patients and suggested that bile acid changing treatment is likely effective to improve IBD-related symptoms [44,48]. Together, it is interesting to speculate that BBG9-1-associated alteration of bile acid metabolism may contribute in part to the improvement of CD patients enrolled although we have no data of intestinal bile acid in this study. In the regard of HDL, it plays a role in not only reverse transport of cholesterol but also anti-inflammatory functions by reprogramming macrophages and/or neutralizing intestinal-leaked LPS [49,50]. From these viewpoints, the reduction of serum HDL might not be beneficial for CD patients in this study. We cannot explain why serum HDL was reduced in CD patients after BBG9-1 treatment. However, we have to re-recognize that our most of CD patients had prior surgery. This may suggest that malabsorption of bile acid is at least affecting bile acid metabolism. Regarding the effect of BBG9-1 on bile acid metabolism including HDL and total cholesterol, we would like to examine in animal model and/or larger scale clinical study in future.

In summary, we have demonstrated that the probiotic BBG9-1 is effective for improvement of IBD-related QOL in patients with qCD and IBS-D-like symptoms, although it did not affect minimal intestinal inflammation, at least in terms of the fecal calprotectin level. This suggests that the observed improvement of IBS-D-like symptoms in those patients was not associated with amelioration of minimal intestinal inflammation. We also clarified that BBG9-1 was effective for reducing anxiety and increasing the abundance of *Bacteroides*, thus ameliorating psychological stress. Since psychological stress is a key factor that can worsen abdominal symptoms in IBS patients, the observed improvement of IBD-related QOL by BBG9-1 may have been be due to relief of anxiety as a result of the increase in beneficial strains. However, it is known that a placebo can also greatly affect abdominal symptoms in patients with IBS [34]. As we did not include a placebo group in this study, we cannot rule out the possibility that the effect of BBG9-1 may have been partially attributable to a placebo effect. In addition, the number of enrolled subjects was small, and a strict run-in period should be set in this study [51]. Although the present study clearly had some limitations, we think that our data warrant a further study in which a placebo group would be included. However, we believe that we have demonstrated for the first time that the probiotic BBG9-1 could be potentially useful for improvement of QOL and/or amelioration of abdominal symptoms in patients with qCD and IBS-D-like symptoms.

## Figures and Tables

**Table 1:** Characteristics of quiescent CD patients with IBS-D-like symptoms.

Age (years)	47.3 ± 10.5 (31–71)
Sex (male/female)	8/3
BMI (kg/m^2^)	21.3 ± 4.0 (15.4–29.8)
Duration of disease (years)	19.6 ± 7.7 (8–33)
Current medications (%)	
5-ASA	6 (54.5)
Steroids	0 (0.0)
Immunomodulator	0 (0.0)
Biologics therapy	1 (9.1)
Elemental diet	10 (90.9)
Prior surgery (%)	8 (72.7)
C-reactive protein (mg/dL)	0.08 ± 0.10 (0.00–0.24)
Crohn’s disease activity index	110.4 ± 25.4 (59.2–141.1)

Results as mean ± SD (range). IBS-D, diarrhea-predominant IBS; BMI, body mass index; 5-ASA, 5-aminosalicylate.

**Table 2:** Effect of BBG9-1 treatment on serum cytokines level in quiescent CD patients with IBS-D-like symptoms.

Cytokines (pg/mL)	Baseline	After 4-Weeks-Treatment	*p* Value
IL-1b	0.01 ± 0.04	0.02 ± 0.07	0.75
IL-2	0.08 ± 0.27	0.00 ± 0.00	0.34
IL-4	0.00 ± 0.00	0.01 ± 0.02	0.34
IL-5	0.00 ± 0.00	0.00 ± 0.00	–
IL-6	0.58 ± 0.71	0.40 ± 0.81	0.53
IL-7	0.39 ± 0.66	0.64 ± 1.31	0.51
IL-8	8.63 ± 6.59	4.64 ± 2.95	0.08
IL-10	0.00 ± 0.00	0.07 ± 0.22	0.35
IL-12	0.06 ± 0.21	0.25 ± 0.83	0.50
IL-13	0.00 ± 0.00	0.00 ± 0.00	–
IL-17	0.07 ± 0.21	0.01 ± 0.02	0.35
G-CSF	0.31 ± 0.89	0.31 ± 0.89	1.00
GM-CSF	0.00 ± 0.00	0.00 ± 0.00	–
IFN-γ	0.35 ± 0.33	0.26 ± 0.21	0.13
MCP-1	7.47 ± 6.71	4.46 ± 3.73	0.048
MIP-1b	16.84 ± 9.91	11.68 ± 5.70	0.08
TNF-α	3.22 ± 4.10	2.74 ± 2.31	0.76

Data are expressed as mean ± SD. IL, interleukin; G-CSF, granulocyte colony stimulating factor; GM-CSF, granulocyte macrophage colony stimulating factor; IFN, interferon; MCP, monocyte chemotactic protein; MIP, macrophage inflammatory protein; TNF, tumor necrosis factor.

## Data Availability

Any data referred to in this work will be available on request.

## Supplementary Materials

The following supporting information can be downloaded at: https://www.mdpi.com/article/10.3390/jcm12103368/s1, Supplementary Table S1: Inclusion and exclusion criteria; Supplementary Table S2: Effect of BBG-9 treatment on blood biochemistry data in quiescent CD patients with IBS-D-like symptoms.
