# Supplementary material for: Effect of Bifidobacterium bifidum G9-1 on the Intestinal Environment and Diarrhea-Predominant Irritable Bowel Syndrome (IBS-D)-like Symptoms in Patients with Quiescent Crohn’s Disease: A Prospective Pilot Study
Source: J Clin Med. 2023 May 9;12(10):3368. doi: 10.3390/jcm12103368 (PMC10219535; doi:10.3390/jcm12103368)
Supplement: Supplementary file 1 [file jcm-12-03368-s001.zip › jcm-2350737-supplementary.pdf]

Supplementary Table S1. Inclusion and exclusion criteria

Inclusion criteria

Subjects who meet all of the following criteria are included in this study:

1. Subjects who had participated in the previous study (Alterations of gut microbiome and mucosal permeability in inactive IBD patients with IBS-like symptom. / UMIN-CTR No. UMIN000041577)
2. Subjects who were suffered by abdominal pain or discomfort continuously (three days or mor within a month), and who show at least two of the following characteristics
   1) Three symptoms were attenuated by defecation
   2) Defecation frequency was changed accompanied with these symptoms (increased or decreased)
   3) Fecal properties were changed accompanied with these symptoms (became soft or hard)
3. Patients with CDAI (Crohn’s Disease Activity Index) of less than 150
4. Patients with CRP of less than 0.3 mg/dL
5. Male and female aged 20 years or older
6. Subjects who give their consent in a written form

Exclusion criteria

Subjects who fall into any of the following criteria are excluded from participating in the study:

1. Subjects who are using any intestinal regulator agents or supplements other than *Bifidobacterium bifidum* G9-1.
2. Subjects with history of myocardial infection, cerebral infarction, or stroke within 12 weeks before giving their consent.
3. Subjects with malignant neoplasm
4. Subjects with history of allergy against *Bifidobacterium*
5. Subjects with other conditions that the responsible investigator or subinvestigators think inappropriate to participate in the study

Supplementary Table S2. Effect of BBG-9 treatment on blood biochemistry data in quiescent CD patients with IBS-D-like symptoms

Blood data	Baseline	After 4-weeks-treatment	*P* value
WBC (/μL)	6802.7 ± 2229.4	6644.5 ± 2404.6	0.64
Hb (g/dL)	14.0 ± 1.6	14.0 ± 1.7	0.86
Platelet (x10^4/mL)	25.5 ± 6.8	25.7 ± 6.6	0.80
Blood sugar (mg/dL)	109.6 ± 23.2	92.7 ± 15.3	0.08
AST (IU/L)	23.7 ± 6.0	25.8 ± 11.1	0.41
ALT (IU/L)	29.5 ± 13.4	29.6 ± 16.9	0.96
γGTP (IU/L)	23.2 ± 16.0	24.3 ± 15.9	0.52
HDL-cholesterol (mg/dL)	69.5 ± 19.7	66.2 ± 17.9	0.024
Total-cholesterol (mg/dL)	173.5 ± 40.7	162.8 ± 32.5	0.022
LDL-cholesterol (mg/dL)	79.2 ± 42.0	77.0 ± 37.1	0.42
TG (mg/dL)	124.0 ± 63.3	105.1 ± 53.8	0.07
UA (mg/dL)	5.5 ± 1.9	5.9 ± 1.3	0.22
BUN (mg/dL)	12.9 ± 2.7	13.4 ± 3.0	0.55
Cr (mg/dL)	0.91 ± 0.23	0.89 ± 0.24	0.25
CRP (mg/dL)	0.08 ± 0.10	0.12 ± 0.16	0.31

Data are expressed as mean ± SD.
